# Hot Deformation Constitutive Analysis and Processing Maps of Ultrasonic Melt Treated A5052 Alloy

**DOI:** 10.3390/ma17133182

**Published:** 2024-06-28

**Authors:** Sun-Ki Kim, Seung-Hyun Koo, Hoon Cho, Seong-Ho Ha

**Affiliations:** 1 NICE LMS Co., Ltd., Yesan 32446, Republic of Korea; 2 Korea Institute of Industrial Technology, Incheon 21999, Republic of Korea

**Keywords:** A5052, ultrasonic treatment, grain refinement, hot deformation, processing map

## Abstract

Hot deformation constitutive analysis and processing maps of ultrasonic melt treated (UST) A5052 alloy were carried out based on a hot torsion test in this study. The addition of the Al–Ti master alloy as a grain refiner with no UST produced a finer grain size than the UST and pure Ti sonotrode. The Al3Ti phase particles in the case of the Al–10Ti master alloy acted as a nucleus for grain refinement, while the Ti atoms dissolved in the melt from the sonotrode were considered to have less of a grain refinement effect, even under UST conditions, than the Al3Ti phase particles in the Al–Ti master alloy. The constitutive equations for each experimental condition by torsion test were derived. In the processing maps examined in this study, the flow instability region was not present under UST in the as-cast condition, but it existed under the no UST condition. The effects of UST examined in this study are considered as (i) the uniform distribution of Ti solutes from the sonotrode and (ii) the reduction of pores by the degassing effect. After the homogenization heat treatment, most instability regions disappeared because the microstructures became uniform following the decomposition of intermetallic compounds and distribution of solute elements.

## 1. Introduction

Al alloys are widely utilized in various industries, such as aerospace and transportation, because they have high strength, low density, corrosion resistance, good electrical conductivity, and thermal conductivity, as well as machinability. Al–Mg alloys belonging to the 5xxx series of Al alloys show an improved strength and corrosion resistance due to the effect of Mg as a crucial strengthening element. A5052 is an Al–Mg alloy supplemented with a small Cr addition, and has moderate strength, good workability, and very good corrosion resistance [1,2,3,4]. The mechanical properties of Al alloys can be controlled by various microstructural characteristics, such as refined grains. In the grain refinement process of Al alloys, the intermetallic compounds such as Al_3_Ti contained in chemical grain refiners, like Al–10Ti and Al–5Ti–1B master alloys, act as nuclei to refine the grains [5]. In a previous study [6], ultrasonic melt treatment (UST) using a commercial purity Ti sonotrode for A5052 alloy melts was examined. As a result, the Ti content dissolved in the A5052 melt was confirmed in the range between 0.06 and 0.07 mass%, which is considered a proper amount to lead to a grain refinement effect. The UST, even only for just a few minutes, refined the columnar zone-prevailing coarse grains into the fine equiaxed grains in the A5052 billet. The refinement by the UST also affected microstructures such as grains, Al–Fe-based particles, dendrites, and pores, leading to a remarkable improvement of mechanical properties in the as-cast condition. Such improvement in billet quality possibly results in an increase in plastic workability.

Hot deformation and dynamic softening affect not only flow stress but also microstructure. In general, the deformation process at high temperatures promotes material flow as well as high dynamic softening. In this process, if the deformation speed is high, the thermal energy generated during the deformation will increase [7,8,9], and many studies in the past have examined the effect of softening by thermal energy generated during deformation. And also, many researchers have proceeded with the premise that the effects must be taken into consideration by analyzing the hot working characteristics [7,8,9,10,11,12]. In general, studies on hot deformation of metallic materials have been carried out through the build-up of processing maps based on the dynamic material model (DMM) under hot working conditions [7,13,14,15,16,17,18,19]. Power dissipation efficiency is known to be related to the microstructure of the material after hot deformation. The stable region in the processing map has been considered to optimize deformation conditions for hot working. The processing maps on the various ferrous and nonferrous materials such as the Al–Si alloy [20], molybdenum [21], NIMONIC 80A [22], Ti–6Al–4V alloy [23], stainless steel [24], and Mg alloys [25,26,27] have been examined. In the case of A5052 alloys, He et al. [4] studied the hot deformation behavior of cast alloy between 300 and 500 °C. In their study, the optimum working parameters in the ranges of strain and temperature for cast 5052 alloy were determined. Son et al. [19] investigated hot deformation characteristics of A5052 modified with CaO added Mg master alloy as an Mg additive. The alloy modification using CaO added Mg was to improve the oxidation resistance of Mg in the A5052 alloy. However, no research on the effect of UST with a Ti sonotrode on the workability of A5052 based on processing maps has been reported. In this study, hot deformation constitutive analysis and processing maps of the ultrasonic melt treated A5052 alloy were carried out based on a hot torsion test.

## 2. Materials and Methods

### 2.1. Fabrication of Materials

**Figure 1:**
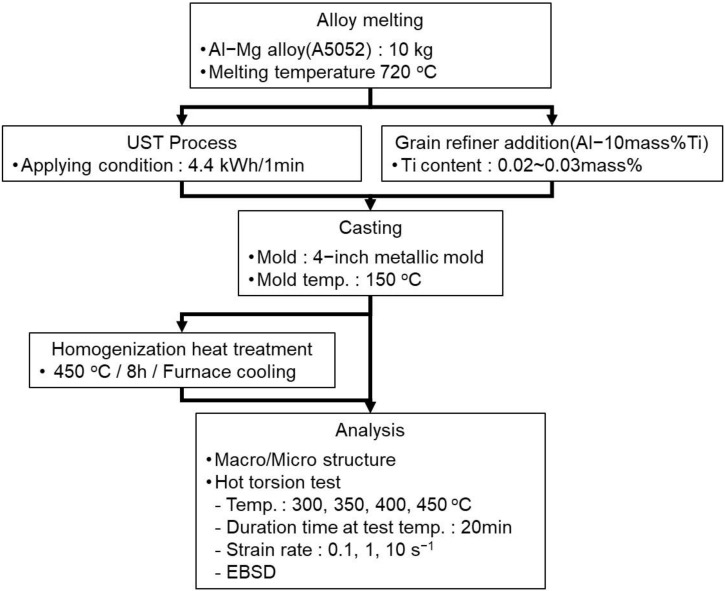
Experimental process conducted in this study.

The composition of the A5052 alloy examined in this study is shown in Table 1. A5052 melts of approximately 10 kg at 720 °C were prepared using an induction furnace. To conduct the UST for the A5052 alloy melts, a 5 kW ultrasonic power supply (Generator, USGC-5-22 MS) and a water-cooled magnetostrictive transducer (MST-5-18) with 20 kHz were used. A commercially pure Ti was employed for the sonotrode material, which was immersed for 3 min to be preheated to the same temperature as that of the A5052 melts. When the temperature of the melts was at 720 °C, the UST was performed at an output of 4.4 kHz for 1 min to 1 min and 30 s. After the UST, the alloy melts were poured into a mold preheated to 150 °C. The addition of Al–10mass%Ti master alloy, which is a common chemical grain refiner, was examined for comparison. The target Ti content was set in the range of 200 to 300 ppm, considering that of the Ti sonotrode in the UST and the minimum content for the grain refinement effect by Ti. The Al–10Ti master alloy ingots were added and held at 720 °C for approximately 10 min. After that, the melts were poured into the same mold as in the UST process and preheated to 150 °C. And then, for comparison between as-cast and as-homogenized conditions, the homogenization heat treatment was performed at 450 °C for 8 h and followed by furnace cooling. The measured compositions of alloy billets with or without the UST process are shown in Table 1. The contents of Mg and Cr, which are the main constituent elements of the A5052 alloy, did not change significantly. Furthermore, the Ti content was found to be 0.025 mass% and 0.02 mass% in the billets with and without the UST, respectively. Therefore, it is thought that the manufactured billets are suitable for use in this study. The experimental process conducted in this study is shown in Figure 1.

The macrostructures and microstructures for the as-cast alloys were compared. The samples for the macrostructures were processed into a disk shape with a thickness of 10 mm and etched using Poulton’s reagent. The microstructures were electrochemically etched by Barker’s regent and observed through a polarizing microscope. The grain size was measured using the linear intercept method specified in ASTM E-112 [28].

### 2.2. Hot Torsion Test

The torsion test specimen was processed into a shape with a length of 20 mm and diameter of 10 mm in the test section from both the as-cast and as-homogenized materials. In the torsion test, the temperature was increased up to the target temperature at 1 °C/s. using an infrared heater. The test began after holding at the target temperature for 10 min to ensure that the temperature of the entire specimen was kept uniform. The test temperatures ranged between 300 and 450 °C, and the strain rate was set from 0.1 to 10 s^−1^. The torsion test continued until the effective strain reached 15 or the specimen fractured. After the test was completed, the specimens were immediately water-cooled to obtain plastic deformation structures. The effective stress (*σ*) and strain (*ε*) of each test specimen were calculated from the torque (*M*) and angular displacement ($\dot{\theta }$) obtained during the torsion test by using the von Mises criterion and the method proposed by Field and Backofen [16]:

(1) $$\theta =\frac{\sqrt{3}(3+p+q)}{2\pi r}$$

(2) $$\varepsilon =\frac{r\theta }{\sqrt{3}L}$$

(3) $$P=\frac{\partial lnM}{\partial ln\dot{\theta }}\;\left|\;\theta ,\;T\right.\;q=\frac{\partial lnM}{\partial ln\dot{\theta }}\;\left|\;\dot{\theta },\;T\right.$$

where *p* is strain rate sensitivity, *q* is the strain hardening coefficient, *r* is the radius of the gauge section, *L* is the gauge length of the sample, and $\dot{\theta }$ is the twist rate.

Microstructures deformed during the hot torsion test were observed and analyzed by electro backscattering diffraction (EBSD) on sections parallel to the torsion axis at the center of the specimens. The samples for EBSD were prepared by electropolishing in a solution of 20% perchloric acid in methanol at −30 °C.

## 3. Results

### 3.1. Macro/Microstructures of Billets

**Figure 2:**
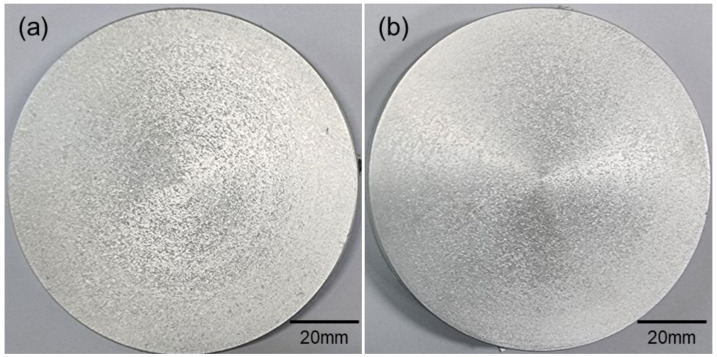
Macrostructures of cross-sectioned disks: (**a**) UST and (**b**) no UST.

**Figure 3:**
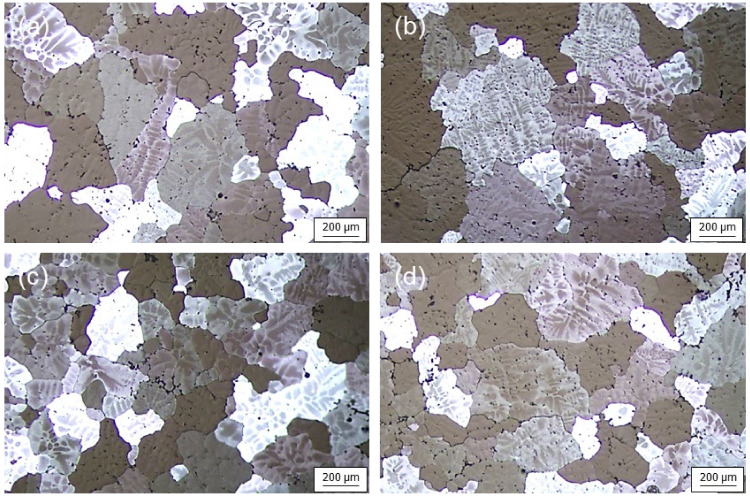
Microstructures of cross-sectioned disks: (**a**) center and (**b**) R/2 with UST, and (**c**) center and (**d**) R/2 with no UST. The average grain sizes are (**a**) 200 μm, (**b**) 230 μm, (**c**) 180 μm, and (**d**) 180 μm, respectively.

The macrostructures of the disks taken from each billet are shown in Figure 2. Equiaxed grains were observed, and cast structures such as chill zones and columnar tablets did not appear in both disks. Therefore, it can be said that the Ti addition of about 200 ppm even without the UST can lead to the significant grain refinement of the as-cast microstructures. Figure 3 shows the microstructures depending on locations in each disk. They exhibited non-uniform structures with a mixture of fine or relatively coarse grains. However, the deviation of grain size was larger in the disk with the UST rather than in that with no UST. As a result of grain size measurement, the average grain sizes of center and R/2 under the UST conditions were 200 μm and 230 μm, and was 180 μm under no UST, respectively. Therefore, it was confirmed that the addition of the Al–Ti master alloy as a grain refiner with no UST produced a finer grain size than the UST and pure Ti sonotrode. In the case of the addition of the Al–10Ti master alloy, the Al_3_Ti phase particles act as a nucleus for grain refinement. However, the Ti atoms dissolved in the melt from the sonotrode are considered to have less of a grain refinement effect, even under the UST conditions, than the Al_3_Ti phase particles in the Al–Ti master alloy.

### 3.2. Constitutive Analysis

The constitutive equation was calculated for each condition as a method for analyzing the relationship among the parameters based on the results of the torsion test. It is generally known that, as the deformation temperature rises, the yield stress and flow stress decrease. Similarly, a decrease in work hardening mainly causes a decrease in stress. When work hardening occurs, the flow stress increases as the strain increases. Therefore, the relationship between the flow stress and the test temperature can be described in order to express the given strain (*ε*) and strain rate ($\dot{\varepsilon }$), which appear in the following Arrhenius-type equation.

$$\sigma_{\varepsilon ,\;\dot{\varepsilon }}=C_{1}\exp(Q/\mathrm{RT})$$

where $\sigma_{\varepsilon ,\;\dot{\varepsilon }}$ is the true stress at the given stain and strain rate, *Q* is the activation energy during deformation, *R* is a gas constant, *T* is the test temperature, and *C*_1_ is a constant.

When this relationship is established, a straight line with a slope of Q/R can be created in a graph with respect to 1/T. However, this equation is only suitable when the mechanism to determine the flow stress works in the same temperatures. The determination of flow stress is limited by one equation at a wide temperature range in which other mechanisms operate. Sellars and McTegart [29] proposed three Arrhenius-type equations that can describe the relationship among the parameters in hot deformation as follows:

(4) $$\dot{\varepsilon }\exp(Q/\mathrm{RT})=A_{1}\sigma_{p}^{n}=Z$$

(5) $$\dot{\varepsilon }\exp(Q/\mathrm{RT})=A_{2}\exp(\beta \sigma_{p})=Z$$

(6) $$\dot{\varepsilon }\exp(Q/\mathrm{RT})=A{\left(\sinh\alpha \sigma_{p}\right)}^{n}=Z$$

where *A*_1_, *A*_2_, *A*, *n′*, *n*, *β*, and *α* (=*β*/*n′*) are material constants (Table 1); and $\dot{\varepsilon }$, *σ_p_*, *Q*, *T*, *R*, and *Z* are strain rate, peak stress, the activation energy for deformation, temperature, gas constant and the Zener–Hollomon parameter, respectively. Equations (4) and (5) break down at high stress and high temperature, respectively. The hyperbolic sine law, Equation (6), is used as a general form suitable for a wide range of applications [30,31].

The material constants were calculated from the effective stress–strain data to build the constitutive equations. Taking natural logarithms on both sides of Equations (4) and (5), the following equations can be obtained:

(7) $$\ln\dot{\varepsilon }=\ln A_{1}+n^{’}\ln\sigma_{p}-Q/\mathrm{RT}$$

(8) $$\ln\dot{\varepsilon }=\ln A_{2}+\beta \sigma_{p}-Q/\mathrm{RT}$$

**Figure 4:**
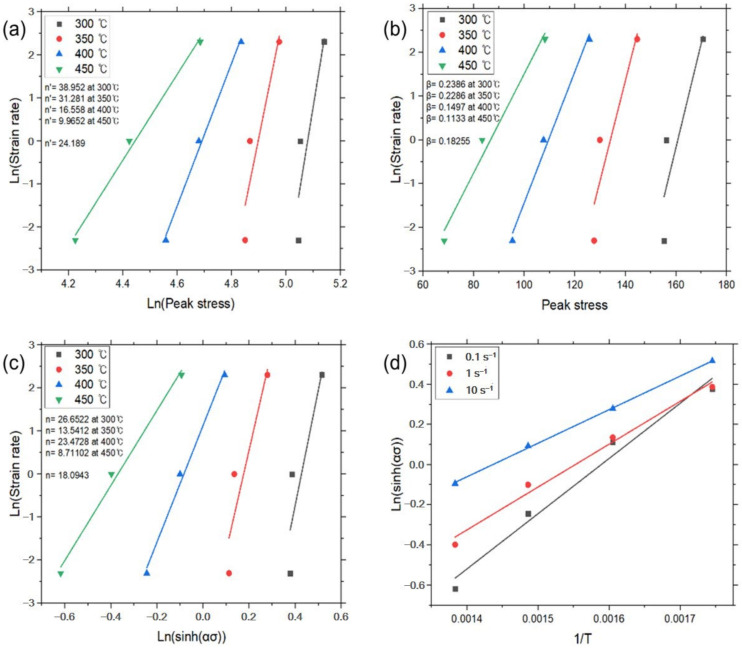
Constitutive analysis of as-cast A5052 alloys with UST according to Equations (4)–(7). (**a**) $\dot{\varepsilon }$–*σ_p_* dependence, (**b**) $\dot{\varepsilon }$–exp(*σ_p_*) dependence, (**c**) $\dot{\varepsilon }$–sinh(*ασ_p_*) dependence, and (**d**) Arrhenius dependence.

And then constants *n′* and *β* were determined from the slope of the plot for ln$\dot{\varepsilon }$–ln*σ_p_* and ln$\dot{\varepsilon }$–*σ_p_*, respectively. The average value of the constants can be determined by the linear fitting method at a given temperature, as shown in Figure 4a,b. The value of *α* (=*β*/*n′*) was calculated by the determined *n’* and *β*. Then, Equation (6) could be rewritten as:

(9) $$\ln\dot{\varepsilon }=\ln A_{1}+n(\sinh\alpha \sigma_{p})-Q/\mathrm{RT}$$

Taking partial differential equations of Equation (8), the activation energy for deformation can be expressed as:

(10) $$Q=R\left[\frac{\partial ln\dot{\varepsilon }}{\partial lnsinh(\alpha \sigma_{p})}\right]\left[\frac{\partial lnsinh(\alpha \sigma_{p})}{\partial (1/T)}\right]=RnS$$

where *s* is a slope of the relationship of ln(sinh*ασ_p_*) – 1/*T* at a given strain rate.

**Figure 5:**
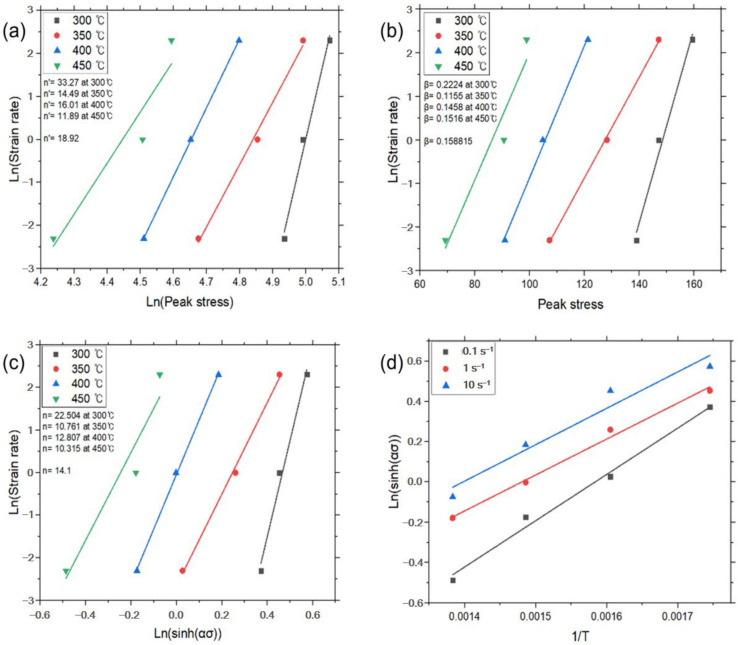
Constitutive analysis of as-cast A5052 alloys with no UST according to Equations (4)–(7). (**a**) $\dot{\varepsilon }$–*σ_p_* dependence, (**b**) $\dot{\varepsilon }$–exp(*σ_p_*) dependence, (**c**) $\dot{\varepsilon }$–sinh(*ασ_p_*) dependence, and (**d**) Arrhenius dependence.

**Figure 6:**
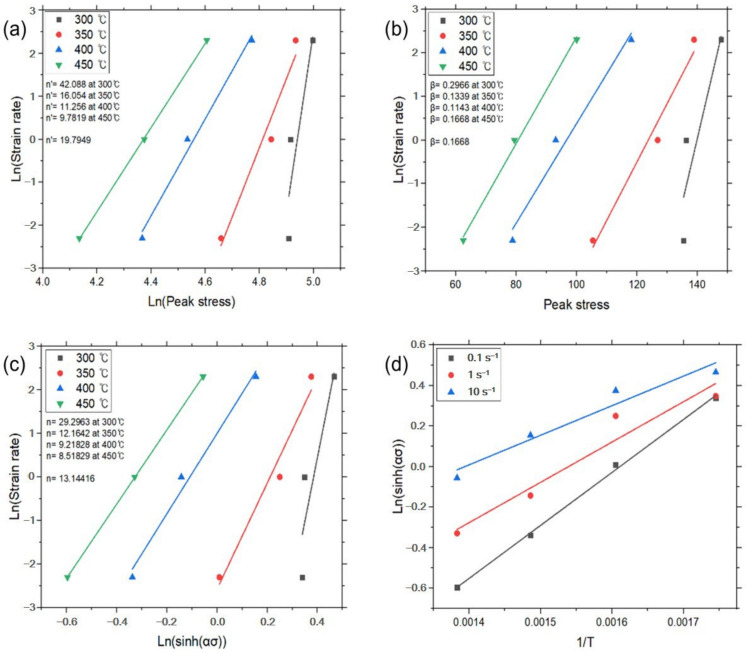
Constitutive analysis of as-homogenized A5052 alloys with UST according to Equations (4)–(7). (**a**) $\dot{\varepsilon }$–*σ_p_* dependence, (**b**) $\dot{\varepsilon }$–exp(*σ_p_*) dependence, (**c**) $\dot{\varepsilon }$–sinh(*ασ_p_*) dependence, and (**d**) Arrhenius dependence.

**Figure 7:**
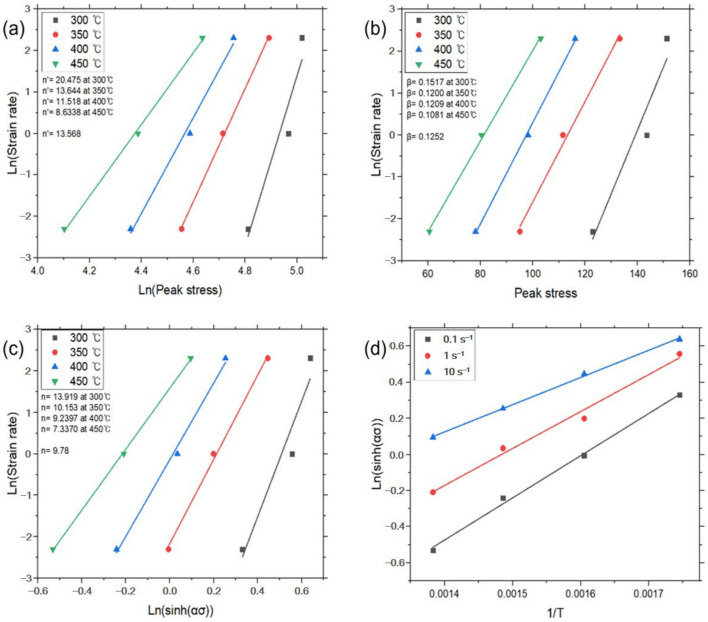
Constitutive analysis of as-homogenized A5052 alloys with no UST according to Equations (4)–(7). (**a**) $\dot{\varepsilon }$–*σ_p_* dependence, (**b**) $\dot{\varepsilon }$–exp(*σ_p_*) dependence, (**c**) $\dot{\varepsilon }$–sinh(*ασ_p_*) dependence, and (**d**) Arrhenius dependence.

The linear relationships of ln$\dot{\varepsilon }$ – ln(sinh*ασ_p_*) and ln(sinh*ασ_p_*) – 1/*T* at a given strain rate were fitted from Figure 4c,d. Constants *n* and *s* were determined by the average value of slope in the plots. Each constant for deriving the constitutive equation was calculated using the above method and is given in Table 2, and the graphs for each experimental condition are shown in Figure 4, Figure 5, Figure 6 and Figure 7.

The constitutive equations of the experimental condition using the derived constants and graphs are shown in Table 3. As a result of examining the activation energy for the constitutive equation, in the case of the as-cast billets, the activation energies under UST and no UST were 284 kJ/mol and 230 kJ/mol, respectively. After the homogenization heat treatment, the activation energies under UST and no UST were 248 kJ/mol and 166 kJ/mol, respectively. The activation energies throughout the entire conditions appear to be significantly high compared with that of pure Al. In general, the activation energy of pure Al is approximately 142 kJ/mol [32,33] to 150 kJ/mol [34], and, when some alloying elements are added, it increases to 200 kJ/mol [35,36,37,38,39,40,41,42,43]. According to the literature [4], the activation energy of the A5052 alloy without Ti was approximately 207 kJ/mol, which is lower than the energies in all conditions except in the homogenization heat treatment under no UST. This possibly occurred because the dislocation was formed during the deformation process in the work-hardening alloy. The deformation resistance is generated and increases depending on the energy storage in the microstructure as the number of dislocations increase [20]. Furthermore, the lower activation energy under no UST means that the energy barrier to dislocation movement is relatively low, making the alloy more favorable for high-temperature plastic working. And also, this possibly occurred because the microstructure under the no UST condition had a relatively small deviation of grain size and a higher fraction of equiaxed grains compared with the UST condition [44,45].

**Figure 8:**
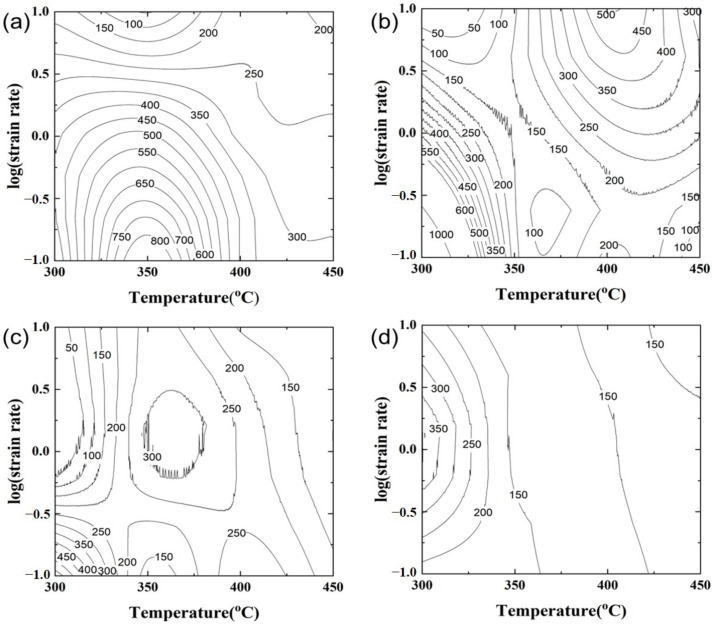
Activation energy maps depending on experimental conditions. (**a**) UST and (**b**) no UST in as-cast condition, and (**c**) UST and (**d**) no UST in as-homogenized condition.

The activation energy distribution depending on strain temperature and rate based on the activation energies determined above was calculated and is shown in Figure 8. The low activation energy in the as-cast and UST conditions corresponds to ≤200 kJ/mol at high strain throughout the entire temperature range. On the other hand, the as-homogenized alloy did not depend significantly on strain and had low activation energies at temperatures around 300 °C and 450 °C. The as-cast alloy with no UST had low activation energies at high strain and around 300 °C, and at low strain and temperatures higher than 350 °C. And also, low activation energy was found at temperatures higher than 350 °C, regardless of the strain rate in the homogenization heat-treated condition.

### 3.3. Processing Map

Power dissipation maps were constructed using experimental data and the principles of the dynamic material model (DMM) [8]. The procedures adopted for the construction of the map are as follows. The relationship between ln$\dot{\varepsilon }$ and ln*σ* at constant temperature and strain was fitted using cubic spline interpolation, and the interpolated curves were fitted by a 3rd order-polynomial. And then, the value of strain rate sensitivity, *m*, was calculated as a function of the strain rate. This procedure was repeated at different deformation temperatures. From the calculated value of *m* at a given temperature and strain rate, power dissipation efficiency was calculated using the following equation:

*η* = *J*/*J_max_* = 2*m*/(*m* + 1) (11)

where *η* is the power dissipation efficiency through microstructural changes.

**Figure 9:**
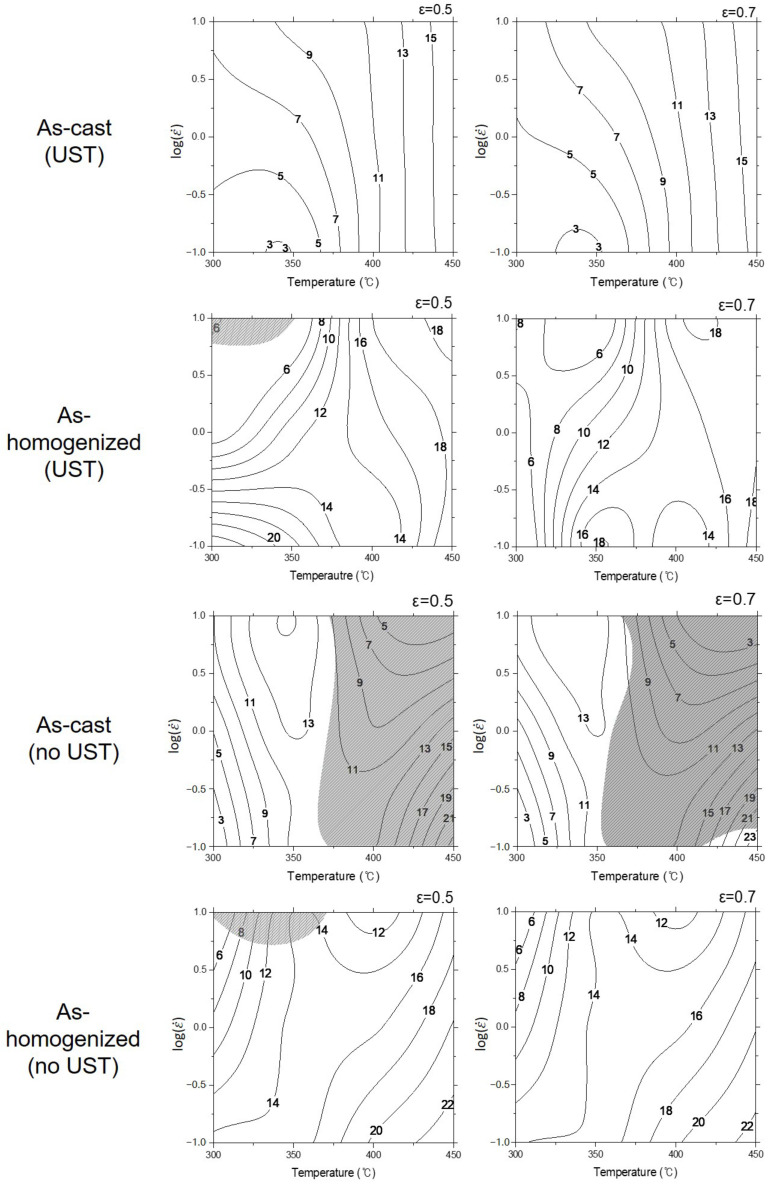
Power dissipation efficiency maps of experimental alloys at a strain of 0.5 and 0.7. The numbers on contour lines represent the power dissipation efficiency calculated from Equation (11).

The calculated power dissipation efficiency for the given strain and temperature for the test conditions in this study is shown in Figure 9. The *η* in the power dissipation efficiency map is represented in a percentage. As shown in Figure 9, the power dissipation efficiency has a lower value under the UST condition than that under no UST in all regions. In addition, the same tendency was observed even after the homogenization heat treatment. Moreover, the flow instability region did not appear at both strain values in the as-cast condition with the UST. However, the wide flow instability regions in the as-cast condition with no UST were observed as the strain and temperature increased. Regardless of UST, the flow instability regions appeared at the low temperatures and high strain rates after the homogenization heat treatment. In particular, the power dissipation efficiency under no UST is high in both as-cast and homogenization conditions at high temperatures and low strain rates. However, in the case of the UST condition, it did not appear to be sensitive to the strain rate as the temperature increased.

### 3.4. EBSD Analysis

**Figure 10:**
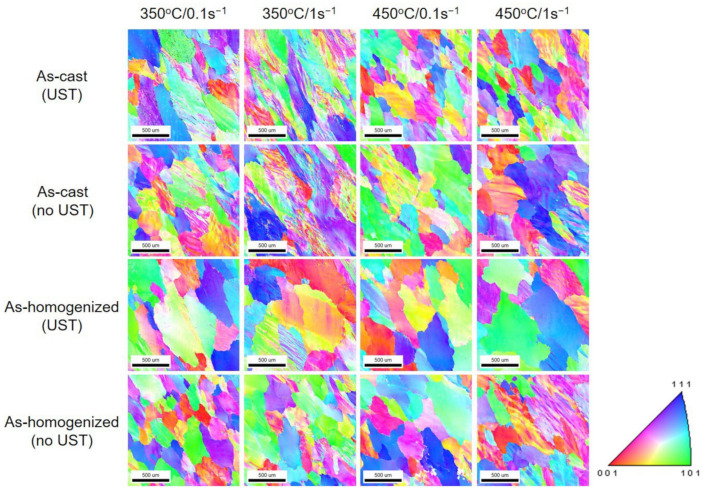
Inverse pole figure map of experimental alloys at 350 °C and 450 °C with 0.1 s^−1^ and 1 s^−1^.

**Figure 11:**
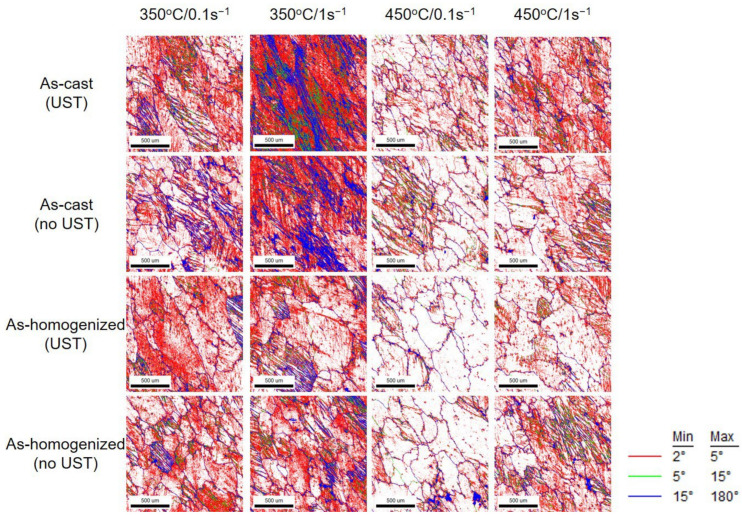
Misorientation maps of the experimental alloys at 350 °C and 450 °C with 0.1 s^−1^ and 1 s^−1^.

The results from the EBSD analysis are given in Figure 10 and Figure 11. They indicate that the presence of a low angle boundary (LAB, 2° < θ < 15°) and high angle boundary (HAB, 15° < θ) increases with increasing strain rates at the same temperature under all experimental conditions. At the same strain rate, the amount of increase in misorientation decreased as the temperature increased. This is possibly associated to stress relief and recrystallization caused by an increase in temperature, although misorientation occurs to form and relieve stress due to plastic deformation. In addition, because Al has low stored energy [46], the energy generated during the deformation should be rapidly eliminated. The thermal energy from such eliminated stored energy possibly induced the recrystallization.

In the as-cast condition, the presence and distribution of LAB and HAB under the UST condition were higher than those under no UST. This is considered to be the different grain size deviation between the UST and no UST conditions, although the average grain size appeared similar in the initial as-cast microstructures. In the case of the UST condition, the grain size deviation was larger than that in the no UST condition, so it may affect the deformation behavior by grain boundary sliding. Therefore, it is thought that the difficulty in deformation led to the higher amounts of LAB and HAB in the torsion test. However, after the homogenization heat treatment, the amounts of LAB and HAB were similar in both the UST and no UST conditions. It is considered that they were not significantly affected by differences in grain size deviation, as the microstructures were stabilized and uniform through the homogenization heat treatment.

**Figure 12:**
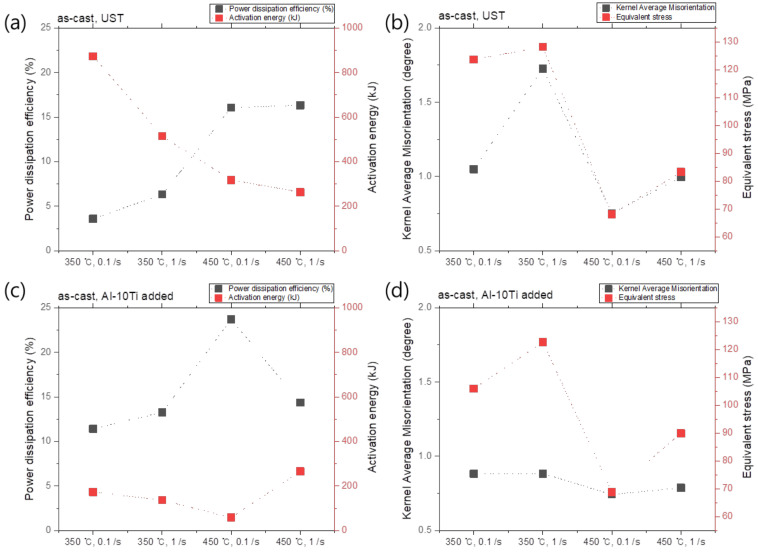
(**a**,**b**) Q-value (activation energy) versus power dissipation efficiency and (**c**,**d**) kernel average misorientation (KAM) versus equivalent stress graphs for (**a**,**c**) UST and (**c**,**d**) no UST in the as-cast condition.

**Figure 13:**
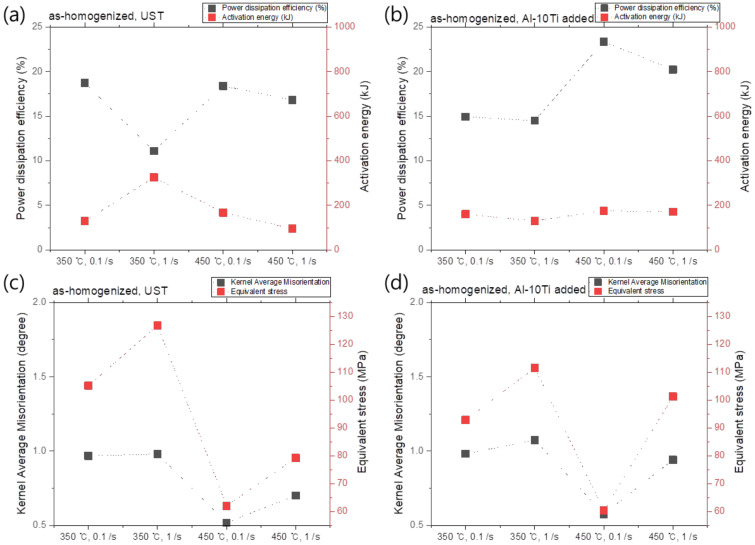
(**a**,**b**) Q-value (activation energy) versus power dissipation efficiency and (**c**,**d**) kernel average misorientation (KAM) versus equivalent stress graphs for (**a**,**c**) UST and (**c**,**d**) no UST in the as-homogenized condition.

The relationships between the power dissipation efficiency (%) and activation energy (Q) under each condition, and the kernal average misorientation (KAM) and equivalent stress, are shown in Figure 12 and Figure 13. In the as-cast condition under the UST, the activation energy increased as the power dissipation efficiency decreased (see Figure 12a). At the same time, the KAM and equivalent stress increased (see Figure 12c). This implies that higher strain energy is required at the same strain, and the activation energy increases with the reduction in power dissipation efficiency, which contributes to changing the microstructure. In addition, a flow instability zone was observed in the region of approximately 400 to 450 °C of the processing map in the case of the no UST condition. In the aforementioned instability region, the misorientation was non-uniformly distributed, which could be associated with the presence of the Al_3_Ti phase by the addition of the Al–10Ti master alloy. In the case of the no UST condition, the Al_3_Ti phase by the addition of the Al–10Ti master alloy as a grain refiner is distributed in melts and remains even after solidification, and begins to decompose at temperatures above 400 °C. However, since the decomposition of the Al_3_Ti phase does not occur within a short period of time, its presence and distribution are possibly not uniform throughout the specimens, thus causing non-uniform deformation.

In the as-cast condition under the UST condition, the power dissipation efficiency was relatively low. However, as the Ti atoms in the Al melt were uniformly distributed and the micropores decreased with the degassing effect during the UST, there should be fewer factors that induced non-uniform deformation. These findings imply that (i) an instability region was not found in all the regions of the processing map, (ii) the deformation at high temperature is not affected by speed, and (iii) the high-speed deformation at high temperature is advantageous. After the homogenization heat treatment, the deformation behavior of the UST and no UST conditions appeared to be similar. Based on these results, it can be said that a high power dissipation efficiency and uniform deformation can be expected in the hot deformation process by maximizing the effect of grain refinement and inducing uniform distribution of Ti solutes when the UST is applied to the Al melt.

## 4. Conclusions

The constitutive equations for each experimental condition by torsion test were derived in this study. From the processing maps, it can be seen that the flow instability region did not exist under the UST in the as-cast condition, but it appeared under the no UST condition. As a result of UST, the uniform distribution of Ti solutes from the sonotrode and the reduction of pores by the degassing effect possibly facilitated the plastic deformation. However, in the case of the no UST condition, the partially undecomposed Al_3_Ti phase and gas pores still remained in the matrix, leading to the creation of instability regions. After the homogenization heat treatment, most instability regions disappeared because the microstructures became uniform following the decomposition of intermetallic compounds and distribution of solute elements. Based on the experimental results in this study, when grain refiners are added to alloys and followed by UST, uniform deformation and high process efficiency can be expected in hot working by maximizing the effect of grain refinement and making the distribution of solutes uniform, and controlling gas pores in the melts.

## Figures and Tables

**Table 1:** Analyzed compositions of A5052 alloy ingot and billets examined in this study.

Sample	Analyzed Compositions (Mass%)								
	Si	Fe	Cu	Mn	Mg	Cr	Zn	Ti	Al
A5052 (Ingot)	0.0524	0.0958	0.0026	0.0048	2.71	0.238	<0.001	0.0028	Bal.
A5052 (UST)	0.0682	0.101	0.0039	0.0057	2.64	0.248	<0.001	0.0255	Bal.
A5052 (no UST)	0.0481	0.111	0.0025	0.0058	2.74	0.248	<0.001	0.0274	Bal.

**Table 2:** Values of each constant depending on temperatures examined in Figure 4, Figure 5, Figure 6 and Figure 7.

	Temp. (°C)	n′	n′ (avg.)	β	β (avg.)	n	n (avg.)
Figure 3	300	38.952	24.189	0.2386	0.18255	26.6522	18.0943
	350	31.281		0.2286		13.5412	
	400	16.558		0.1497		23.4728	
	450	9.9652		0.1133		8.71102	
Figure 4	300	33.27	18.92	0.2224	0.158815	22.504	14.1
	350	14.49		0.1155		10.761	
	400	16.01		0.1458		12.807	
	450	11.89		0.1516		10.315	
Figure 5	300	42.088	19.7949	0.2966	0.1668	29.2963	13.14416
	350	16.054		0.1339		12.1642	
	400	11.256		0.1143		9.21828	
	450	9.7819		0.1668		8.51829	
Figure 6	300	20.475	13.568	0.1517	0.1252	13.919	9.78
	350	13.644		0.1200		10.153	
	400	11.518		0.1209		9.2397	
	450	8.6338		0.1081		7.3370	

**Table 3:** Constitutive equation for each condition.

UST	As-cast	$\dot{\varepsilon }=1.64\times 10^{23}{\left(\sinh0.0075\sigma_{p}\right)}^{16.9}\exp\left(-\frac{284,308}{RT}\right)$
	As-homogenized	$\dot{\varepsilon }=1.26\times 10^{18}{\left(\sinh0.0084\sigma_{p}\right)}^{14.1}\exp\left(-\frac{230,154}{RT}\right)$
No UST	As-cast	$\dot{\varepsilon }=1.9\times 10^{20}{\left(\sinh0.0084\sigma_{p}\right)}^{13.25}\exp\left(-\frac{248,654}{RT}\right)$
	As-homogenized	$\dot{\varepsilon }=9.614\times 10^{12}{\left(\sinh0.0092\sigma_{p}\right)}^{9.78}\exp\left(-\frac{166,141}{RT}\right)$

## Data Availability

The raw data supporting the conclusions of this article will be made available by the authors on request.
